# Production and Immunological Characterization of scFv Specific to Epitope of *Opisthorchis viverrini* Rhophilin-Associated Tail Protein 1-like (OvROPN1L)

**DOI:** 10.3390/tropicalmed8030160

**Published:** 2023-03-06

**Authors:** Amornrat Geadkaew-Krenc, Dawid Krenc, Jeeraphong Thanongsaksrikul, Rudi Grams, Wansika Phadungsil, Kittirat Glab-ampai, Pathanin Chantree, Pongsakorn Martviset

**Affiliations:** 1 Graduate Program in Biomedical Sciences, Faculty of Allied Health Sciences, Thammasat University, Pathumthani 12120, Thailand; 2 Thammasat University Research Unit in Parasitic Diseases, Pathumthani 12120, Thailand; 3 Chulabhorn International College of Medicine, Thammasat University, Pathumthani 12120, Thailand; 4 Center of Research Excellence in Therapeutic Proteins and Antibody Engineering, Department of Parasitology, Faculty of Medicine Siriraj Hospital, Mahidol University, Bangkok 10700, Thailand; 5 Department of Preclinical Science, Faculty of Medicine, Thammasat University, Pathumthani 12120, Thailand

**Keywords:** *Opisthorchis viverrini*, OvROPN1L, single-chain variable fragment (scFv), phage display

## Abstract

(1) Background: *Opisthorchis viverrini* is a significant health problem in the Mekong subregion of Southeast Asia, causing aggressive cholangiocarcinoma. Current diagnostic procedures do not cover early diagnosis and low infection. Hence, an effective diagnostic tool is still required. Immunodiagnosis seems promising, but attempts to generate monoclonal antibodies have not yet been successful. This study aims to develop a single-chain variable antibody fragment (scFv) against Rhophilin-associated tail protein 1-like (ROPN1L), the sperm-specific antigen of adult *O. viverrini*, which has not been reported elsewhere. (2) Methods: The target epitope for phage screening was L3-Q13 of OvROPN1L, which showed the highest antigenicity to human opisthorchiasis analyzed in a previous study. This peptide was commercially synthesized and used for phage library screening. The isolated phage was produced in a bacterial expression system and tested for specificity in vitro and in silico. (3) Results: One of fourteen phages, named scFv anti-OvROPN1L-CL19, significantly bound to rOvROPN1L compared with non-infected hamster fecal extracts. This phage clone was successfully produced and purified using Ni-NTA chromatography. Indirect ELISA demonstrated that scFv anti-OvROPN1L-CL19 has a high reactivity with *O. viverrini*-infected hamster fecal extracts (12 wpi, *n =* 6) in comparison with non-infected hamster fecal extracts (0 wpi, *n* = 6), while the polyclonal rOvROPN1L antibodies did not show such a difference. Molecular modeling and docking confirmed our in vitro findings. (4) Conclusion: scFv anti-OvROPN1L-CL19 could be used as an effective material for developing *O. viverrini*-immunodiagnostic procedures in the future.

## 1. Introduction

*Opisthorchis viverrini* (*O. viverrini*), the human liver fluke, is a parasitic pathogen belonging to the family Opisthochiidae, together with two other known parasitic flatworms, *Opisthorchis felineus* and *Clonorchis sinensis. Opisthorchis viverrini* occurs throughout the Mekong subregion of Southeast Asia, whereas *O. felineus* is mainly found in Europe and *C. sinensis* is concentrated in East Asian countries [1,2]. *Opisthorchis viverrini* infection causes mild to severe symptoms, including abdominal discomfort, fever, jaundice, biliary obstruction, cholangitis, and cholangiocarcinoma (CCA) at the end stage [1,3]. The relationship between *O. viverrini* infection and CCA is highly concerning because CCA is an aggressive cancer with a poor prognosis and a high mortality rate [2,4]. For these reasons, early diagnosis is still required to limit severe complications.

The gold standard for diagnosing *O. viverrini* infection is stool examination using a light microscope with a simple wet smear and formalin-ethyl acetate concentration technique (FECT) [5,6]. However, these methods require the professional experience of the investigator to differentiate O. *viverrini* eggs from other parasite eggs, and they do not enable the diagnosis of light infections with a low level of infection [7,8]. Molecular diagnosis is another possibility; several targets have been reported, such as NADH dehydrogenase (NAD) subunits [9,10,11], internal transcribed spacer (ITS-1 and ITS-2) [12,13], and cytochrome c oxidase 1 (cox1) [13,14]. Various methods have been introduced, such as conventional PCR, qPCR, and LAMP, but the variation in sensitivity and specificity of the target genes is still questionable. Moreover, there are many limitations, such as the instrumental requirement and the price of detection reagents [15,16,17].

Immunodiagnosis is another platform for detecting *O*. *viverrini* infection [18,19]. Among the reported targets, proteins in excretory/secretory (ES) products are highly potentiating [19,20,21]. In addition, parasite ES protein detection has been established for serum and urine specimens [22,23,24]. However, methods for detecting ES proteins that mainly comprise enzymes and enzyme inhibitors remain in development due to the stability of detection targets and cross-reactivities as critical factors [24,25]. Proteins in the eggs, especially the eggshell, are another promising target; a glycine–tyrosine-rich eggshell protein (OvESP) has been characterized, but, unfortunately, its sensitivity is varied [26].

Spermatogenesis is one of the most important biological processes in the life cycle of adult *O. viverrini* inhabiting the host. Previous reports have mentioned that this sperm-associated protein could be used as a detection target for parasitic infection, especially in egg-containing specimens [27,28]. Rhophilin, a RhoA-binding protein, is a sperm-specific protein that is highly expressed in the fibrous sheath of spermatozoa [28,29,30]. In mammals, Rhophilin-1 and its orthologues contain the N-terminal domain, ROPN1, which interacts with A-kinase anchor protein 3 (AKAP3) in both GDP- and GTP-bound RhoA in vitro. ROPN1 binding regulates the actin cytoskeleton by interacting with various downstream molecules [31,32]. Its N-terminal domain is interesting since it is found in only a few proteins [28,33]. The rhophilin-associated tail protein 1-like (ROPN1L) has been molecularly characterized in *O. viverrini* [34,35]. Its best immunogenicity has been identified at the N-terminal region, L3-Q13, established in the serum of infected animals and humans [34]. Interestingly, as OvROPN1L is expressed in young parasites, starting from 2-week-old juveniles [35], it could be a potential early diagnostic target for *O*. *viverrini* infection. As OvROPN1L was found in sperm, a coproantigen could be a source of antigen detection. Therefore, the principal objective of this current study is to produce the single-chain variable fragment (scFv) specifically for OvROPN1L using phage display technology. Moreover, preliminary data on testing the generated scFv with infected and non-infected hamster fecal extracts are provided.

## 2. Materials and Methods

### 2.1. Production of Recombinant Protein OvROPN1L

The recombinant protein OvROPN1L (rOvROPN1L) was produced as described in a previous study [34]. Briefly, the hamsters were infected with *O. viverrini* metacercariae collected from the naturally infected fish. Mature parasites were collected from the infected hamster livers and bile ducts and used for total RNA isolation in TRIzol. The OvROPN1L cDNA fragment was generated using PCR, inserted into the pGEM-T easy vector (Promega, Madison, WI, USA), and subcloned into the pCold™ TF DNA (TaKaRa, Shiga, Japan) expression vector. rOvROPN1L was produced in BL21 E. coli as an expression host strain, coupled with trigger factor (TF) fusion protein using terrific broth and isopropyl β-D-1-thiogalactopyranoside (IPTG) induction. rOvROPN1L was purified under native conditions via gravity flow using a Ni-NTA agarose column (QIAGEN, Hilden, Germany). The TF fusion protein was removed by factor Xa (Promega, Madison, WI, USA). The rOvROPN1L concentration was measured using a Bradford protein assay (Biorad, Hercules, CA, USA).

### 2.2. Polyclonal Antibody Production

Anti-rOvROPN1L antibodies were produced in two female BALB/c mice as previously described [36,37]. At 6 to 8 weeks of age, the mice were immunized at 2-week intervals via three intraperitoneal injections. Pre-immunization sera were collected a week before the first injection. Purified rOvROPN1L was used as an immunogen (10–20 μg per mouse). The final sera (second-boosted sera) were collected two weeks after the last injection by cardiac puncture. Specific antibody titers were determined using indirect ELISA.

### 2.3. Preparation of Coproantigens

The coproantigens were prepared from the feces of *O*. *viverrini*-infected hamsters, as previously described [38]. Six female-Syrian golden hamsters were infected with 100 *O. viverrini* metacercariae each via gastric intubation. Feces were collected at 0 and 12 weeks post-infection (wpi) using sterile plastic containers. The feces were resuspended and homogenized with PBS containing 0.05% Tween-20 (1 g of feces per 3 mL of PB) using a homogenizer (T 10 basic ULTRA-TURRAX^®^, IKA, Staufen, Germany). The homogenate was centrifuged at 3300× *g*, for 10 min, and the supernatant was collected as the coproantigens.

### 2.4. Immobilization of Antigens

The target OvROPN1L peptide, consisting of L3-Q13 (LVNDPYYCHEQ), was selected from our previous study [34]. This peptide was synthesized and conjugated with biotin at the N-terminus by ChinaPeptides (Shanghai, China). The synthesized peptide was dissolved in PBS at pH 7.4 to a final concentration of 2 µg/mL. Thirty micrograms of streptavidin magnetic beads (Pierce™ Streptavidin Magnetic Beads, Thermo Fisher Scientific, Rockford, IL, USA) were washed three times with PBS containing 0.05% Tween-20 (PBS-T) using DynaMag™-Spin Magnet (Invitrogen, Thermo Fisher Scientific, Carlsbad, CA, USA) to separate the beads. Then, the beads were mixed with PBS containing 3% bovine serum albumin (BSA) and incubated on a rotator at 25 °C for 1 h to block the empty surfaces. The beads were washed three times with PBS-T and collected using the DynaMag Spin. As indicated in the instruction manual, the maximum binding capacity of 30 µg streptavidin magnetic beads is approximately 6.6 × 10^12^ molecules of biotinylated antigen. The beads were subsequently mixed with 1 µg OvROPN1L peptide dissolved in 500 µL PBS (approximately 4.36 × 10^14^ molecules) and incubated at 25 °C for 12 h on a rotator to immobilize the antigen on the streptavidin magnetic beads. The excess peptides were removed by washing the beads three times with PBS-T 0.05%. The antigen-bound beads were magnetically separated, resuspended in PBS, and maintained at 4 °C before use in the biopanning step. The ratio of peptide to streptavidin magnetic beads and the immobilization conditions of the antigen were derived via optimization.

For subtraction of nonspecific binders, 5 µg of non-infected hamster fecal extract (coproantigens at 0 wpi) and biotin (BAC-SulfoNHS, Sigma Aldrich, Darmstadt, Germany) were separately mixed with carbonate coating buffer (pH 9.6) in a final volume of 100 μL, coated in wells of an ELISA plate, and incubated at 37 °C for overnight until dry. The wells were washed three times with PBS-T and blocked with 3% BSA in PBS at 37 °C, for 1 h, in a moisture chamber. Excess antigens were removed by washing with PBS-T. The wells were filled with PBS and kept at 4 °C before use in the biopanning step.

### 2.5. Biopanning

In this study, a library of bacteriophages (phages) displaying a repertoire of naïve mouse single-chain variable fragment (scFv) antibodies, previously constructed and described [39], was used to select scFv antibodies specific to the OvROPN1L peptide. The scFv-phage library had approximately 1.3 × 10^8^ antibody diversity and ~1.4 × 10^12^ colony-forming units (CFU)/mL. The input amounts of scFv phages were approximately 10^7^ CFU. Firstly, scFv phages that bound nonspecific antigens were absorbed from the input antibody library via incubation with non-infected hamster fecal extract and biotin. Then, the unbound library was selected for parasitic peptide-bound scFv phages by incubating with peptide-bound magnetic beads at 25 °C for 1 h on a rotator. The unbound phages were washed out with PBS-T and eliminated using magnetic separation. The parasitic peptide-bound scFv phages were eluted from the magnetic beads and allowed to infect log-phase XL-1 Blue *E. coli*, which was used to select transformed bacterial colonies harboring recombinant phagemids, carrying the scfv inserted sequences by PCR, as previously described [39]. Monoclonal scFv phages were produced from the selected bacterial clones, and the titers of the rescued scFv phages were determined and normalized using CFU count [39].

### 2.6. Screening of the scFv Phages Binding with Full-Length rOvROPN1L and Non-Infected Coproantigen

Indirect ELISA was used to determine the antigen-binding ability of the rescued scFv phages. One microgram of rOvROPN1L and non-infected hamster fecal extract (0 wpi) was mixed (1:5 dilution in coating buffer) with carbonate–bicarbonate coating buffer (pH 9.6), coated in the wells of an ELISA plate, and incubated at 37 °C overnight until dry. Afterwards, the wells were washed three times with PBS-T and incubated with 3% BSA in PBS at 37 °C for 1 h. Individual scFv-phage clones were normalized to 10^8^ CFU via dilution with PBS-T and incubation with antigen-coated wells at 37 °C for 1 h. The wells were incubated with an anti-rOvROPN1L antibody (1:512,000 dilution), LB broth, and M13KO7 wild-type phages that served as positive, background, and negative controls, respectively. The wells were washed five times with PBS-T to remove excess contents. Then, wells of the tests and background and negative controls were mixed with a 1:6000 diluted mouse anti-M13 antibody solution (GE Healthcare, Chicago, IL, USA). Finally, wells of tests and all controls were mixed with 1:5000 diluted goat anti-mouse horseradish peroxidase-conjugated antibody (SouthernBiotech, Birmingham, AL, USA), followed by ABTS substrate (SeraCare, Milford, MA, USA). The scFv phages that yielded OD450 nm values with the recombinant protein that were at least twice as significant as the background controls were selected.

### 2.7. Production of Soluble Anti-OvROPN1L scFv Antibody

The soluble scFv was produced by subcloning the *scfv* coding sequence of the selected clone from the pSEX81 phagemid into the pOPE101 plasmid (Progen Biotechnik GmbH, Heidelberg, Germany) via NotI/NcoI restriction sites, as previously described [39]. The coding sequence of the inserted *scFv* was verified by DNA sequencing and predicted for the complementarity determining regions (CDRs) and immunoglobulin framework regions (FRs) by the IMGT/V-QUEST tool (the International ImMunoGeneTics Information System, IMGT^®^, Montpellier, France) [40]. Verified recombinant plasmids were introduced into XL-1 Blue *E. coli* using a heat-shock method. Soluble scFv was produced in the transformed *E. coli* grown under isopropyl β-D-1-thiogalactopyranoside (IPTG) induction and purified using Ni–NTA affinity chromatography under denaturing conditions, then step-wise dialyzed in Urea-PBS buffer.

The purified soluble scFv was verified via Western blot analysis using mouse monoclonal anti-c-Myc (BioLegend, San Diego, CA, USA). The antigen–antibody immune complexes were detected using a goat anti-mouse alkaline phosphatase-conjugated antibody (SouthernBiotech, Birmingham, AL, USA), followed by BCIP/NBT (SeraCare, Milford, MA, USA).

### 2.8. Binding Assessment of Anti-OvROPN1L scFv Antibody with Coproantigens

The soluble scFv anti-OvROPN1L and anti-OvROPN1L antibodies were tested with coproantigens from the *O. viverrini*-infected hamster feces at 0 and 12 weeks post-infection (wpi), *n* = 6, by indirect ELISA, as described in the previous section.

### 2.9. Molecular Modeling and Docking

The deduced amino acid sequences of the generated scFv and OvROPN1L were individually submitted online at AlphaFold2 [41] to model the 3D structure of each protein. Both models were docked with the free online server HADDOCK version 2.4 [42]. The best-docked model was selected using the HADDOCK and PRODICGY webservers [43,44] and the interaction was analyzed using the Discovery Studio Visualizer 2021, version 21.

### 2.10. Statistical Analysis

**Figure 1:**
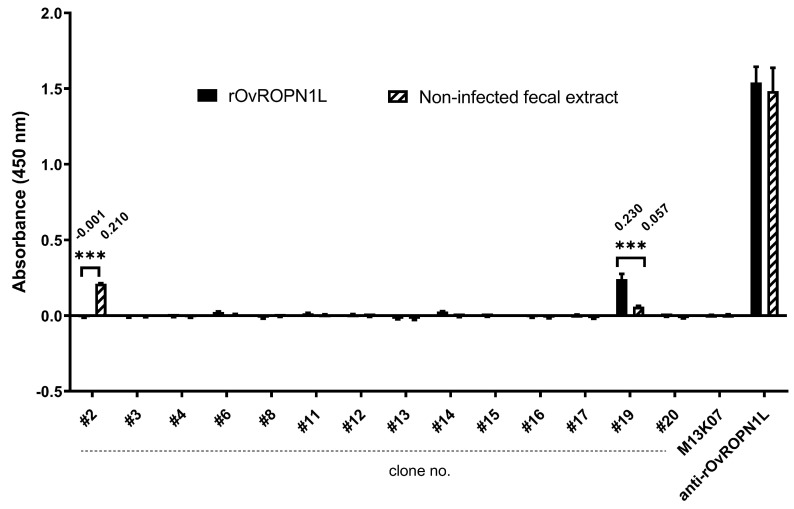
Screening of fourteen *Escherichia coli* clone-isolated scFv phages with rOvROPN1L and pooled non-infected fecal extract (0 wpi coproantigen) using indirect ELISA. Results are presented as mean ± SD, with the triple asterisks (***) indicating statistical significance (*p* < 0.001) calculated via *two-way ANOVA* analysis.

The statistics of the indirect ELISA results, as shown in Figure 1 and Figure 3, were evaluated using a *two-way ANOVA* and Wilcoxon matched-pairs signed rank test, respectively, in GraphPad Prism 9.5. *Two-way ANOVA* was used to estimate the means of absorbance values from two independent groups (testing with rOvROPN1L and non-infected fecal extract, Figure 1). The Wilcoxon matched-pairs signed-rank test was used for non-parametric values to test for the difference in the median of absolute/relative absorbance values from the matched samples (0 and 12 wpi samples are from the same hamsters).

## 3. Results

### 3.1. scFv Phages Specifically Bound to Recombinant OvROPN1L

Phage library screening yielded fourteen XL1-blue *E. coli* clones carrying phagemids with *scFv* inserts detected using direct colony PCR. The progenies of scFv phages were isolated, rescued, and tested for binding to the rOvROPN1L. Pooled non-infected fecal extract (coproantigen at 0 wpi, *n =* 6) was a background control, M13KO7 wild-type phage was a negative control, and mouse anti-rOvROPN1L antibody was a positive control. All clones of scFv phages and M13KO7 wild-type phages were normalized based on the same CFU. Among the fourteen clones, the results demonstrate that only one clone, clone no. 19, is significantly bound to rOvROPN1L, compared with non-infected fecal extract (Figure 1). In contrast, the mouse anti-rOvROPN1L antibody strongly reacted with both rOvROPN1L and non-infected fecal extract, indicating that the anti-rOvROPN1L antibody generated a high background in the fecal extract.

### 3.2. scFv Specific to OvROPN1L Has Been Successfully Cloned

**Figure 2:**
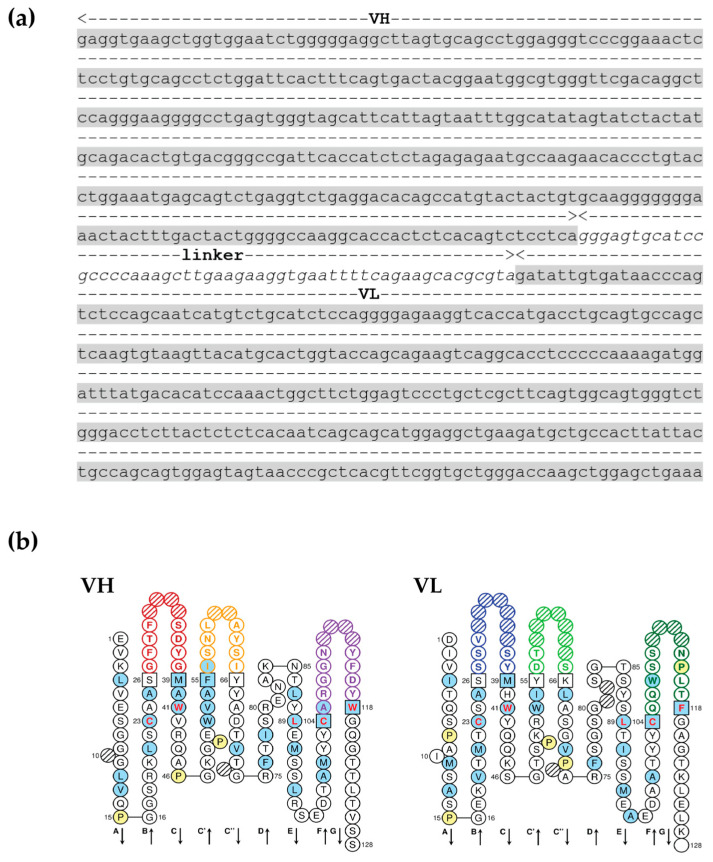
DNA sequence analysis of scFv anti-OvROPN1L-CL19 from recombinant pOPE101 plasmid. (**a**) Nucleotide sequence and immunoglobulin domain prediction of scFv anti-OvROPN1L-CL19. The scFv anti-OvROPN1L-CL19 had heavy-chain (VH) and light-chain (VL) variable domains connected by a linker. (**b**) The 2D graphical representations of VH and VL protein domains of scFv anti-OvROPN1L-CL19 were drawn by IMGT/Collier-de-Perles.

The *scFv* DNA fragment of clone no. 19 was digested and subcloned into the pOPE101 expression vector. Positive transformants were selected by direct colony PCR, and the DNA was sequenced. The DNA sequence of scFv originating from clone no. 19 against OvROPN1L (scFv anti-OvROPN1L-CL19) was analyzed for immunoglobulin properties. The results demonstrate that scFv anti-OvROPN1L-CL19 contains a complete variable domain for both heavy-chain (VH) and light-chain (VL) antigen-binding fragments (Figure 2).

### 3.3. Soluble Recombinant scFv Anti-OvROPN1L-CL19 Was Successfully Produced and Preliminarily Tested against Coproantigens

**Figure 3:**
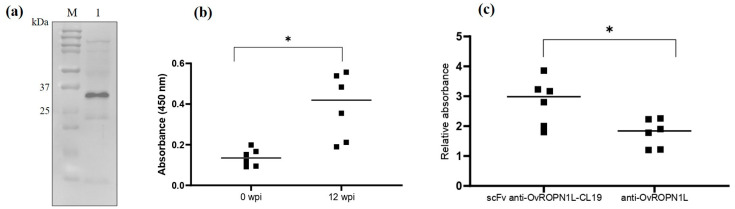
(**a**) Purified scFv anti-OvROPN1L-CL19 at the predicted molecular weight (26–28 kDa); M = broad range protein ladder; lane 1 purified scFv anti-OvROPN1L-CL19; (**b**) Indirect ELISA result of scFv anti-OvROPN1L-CL19 with coproantigens at 0 and 12 wpi of *O. viverrini*-infected hamsters (*n =* 6); (**c**) The relative absorbance values (OD_12 wpi_/OD_0 wpi_) of scFv anti-OvROPN1L-CL19 and anti-OvROPN1L antibody to coproantigens of *Opisthorchis viverrini*-infected hamsters (*n =* 6, each). The asterisk (*) represents statistical significance (*p* < 0.05) calculated by Wilcoxon matched-pairs signed-rank test.

Soluble scFv anti-OvROPN1L-CL19 was successfully produced and then verified using Western blot detection, as shown in Figure 3a. The predicted molecular weight of scFv anti-OvROPN1L-CL19 was 26–28 kDa.

OvROPN1L detection in 0 and 12 wpi coproantigen solutions with purified soluble scFv anti-OvROPN1L-CL19 was tested via indirect ELISA. As expected, scFv anti-OvROPN1L-CL19 significantly reacted with coproantigens at 12 wpi compared with 0 wpi coproantigens (*p =* 0.0313), as shown in Figure 3b. In contrast, the anti-rOvROPN1L polyclonal antibody solution showed no significant reactivity difference between 0 and 12 wpi coproantigens (data not shown). The relative absorbance (OD_12 wpi_/OD_0 wpi_) of scFv anti-OvROPN1L-CL19 and anti-OvROPN1L antibodies, shown in Figure 3c, indicated a significantly higher specific reactivity of scFv anti-OvROPN1L-CL19 (*p* = 0.0414).

### 3.4. Molecular Docking Confirmed the Binding of scFv Anti-OvROPN1L-CL19 and OvROPN1L

**Figure 4:**
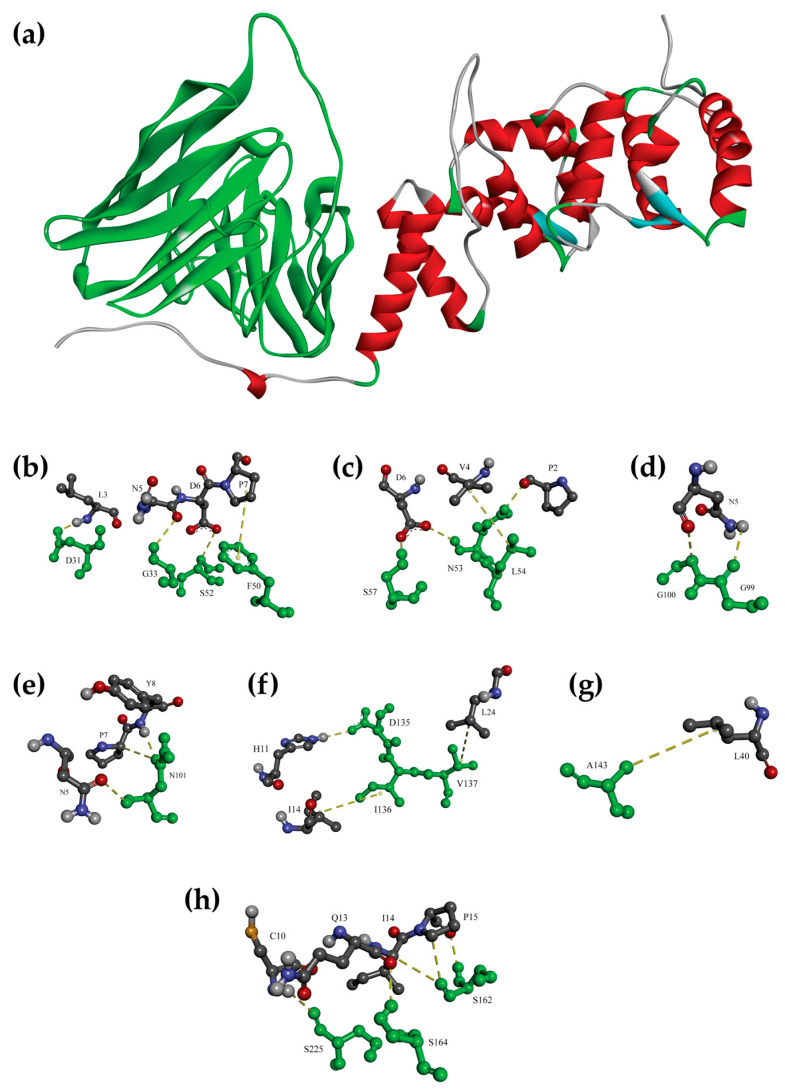
Computerized interactions between the modeled OvROPN1L (red, mainly alpha-helical) and scFv anti-OvROPN1L-CL19 (green, mainly beta-sheets), as determined by molecular modeling and docking (**a**). The interacting amino acids of the antigen-binding domains of scFv anti-OvROPN1L-CL19 (green sticks) and the OvROPN1L peptide (grey sticks) are shown below (**b**–**h**). Interaction details are listed in Table 1.

The molecular 3D modeling and molecular docking of OvROPN1L and scFv anti-OvROPN1L-CL19 were performed, and the results are shown in Figure 4. These results suggest that the modeled scFv anti-OvROPN1L-CL19 interacts with the modeled OvROPN1L through hydrogen, π-alkyl, and hydrophobic non-covalent bonds and that both the VH and VL domains are involved. The predicted binding free energy (Δ*G*) and dissociation constant (K_d_) were −14.2 kcal mol^−1^ and 0.039 nM, respectively. The interacting amino acids of OvROPN1L and scFv anti-OvROPN1L-CL19, as well as the types of molecular interactions, are listed in Table 1.

## 4. Discussion

Infection caused by the human liver fluke, *O*. *viverrini*, is a significant health problem in Mekong sub-region countries. Long-term infection can lead to cholangiocarcinoma (CCA), a highly progressive cancer with a poor prognosis and high mortality [2]. Therefore, an effective and practical early diagnosis procedure would be considerably beneficial in terms of public health.

Monoclonal antibodies, MoAbs, are an effective tool for the immunodiagnosis of many diseases, especially infectious diseases. The detection of specific proteins or target molecules with little cross-reactivity is ideal. For *O. viverrini* infections, several monoclonal antibodies have been developed along with other procedures [9,14,45,46,47]. Most researchers consider secreted antigens in fecal specimens, coproantigens, as the best specimens containing parasite proteins. Excretory/secretory (ES) antigens, such as 89 kDa glycoprotein and 73 kDa protein, were the first target group of monoclonal antibody development as they are continuously released during an infection [46]. Nevertheless, no MoAb is currently solely used in the diagnosis due to MoAbs obtained by different methods showing variable sensitivities. Monoclonal antibody-based ELISA and DNA hybridization methods reportedly show lower or comparative sensitivity to classical parasitological methods [46,48]. In contrast, the sensitivity of the immunoprecipitation method was only 50% with cross-reactivities, as well as known (*Clonorchis sinensis*) and unknown parasites [49]. The 89 kDa glycoprotein was tested by another group using ELISA, and their results illustrate a sensitivity higher than for immunoprecipitation but comparable to stool examination [50]. MoAb dot ELISA was used with an affinity-purified oval antigen from the adult parasite. The in vitro result had 100% sensitivity, but the obtained MoAb was not tested with natural specimens, such as feces or urine [51].

The study of another specimen, urine, is also being conducted. MoAb has been developed and tested in urine specimens against the parasite ES protein: cysteine protease [52]. The sensitivity of the detection of parasite antigens in urine samples was comparable to stool examination and lower when compared to the FECT method [23,52].

In this study, we generated a MoAb against the sperm-specific antigen, OvROPN1L, an aspect that has not been explored elsewhere. OvROPN1L has potential immunogenicity, as previously reported [34,35]. This is the first report of MoAb production specific to an antigen from *O. viverrini* sperm. The phage display technology that we used in this study differs from the methods of previous studies that primarily employ a hybridoma technique. Phage display technologies enable the rapid production of MoAb with a high specificity, lower toxicity, and no requirement for antibody-producing animals [53]. Moreover, the scFv-phage library was determined to contain a broad diversity of *scFv* sequences, from which scFv antibodies to the peptide epitopes located on virion protein 2 of enterovirus A71 and Dia blood group antigen were successfully selected [39,54]. In the current study, the specific reactivity of the selected target epitope for scFv-phage screening was verified in *O. viverrini*-infected human serums and other helminth-infected human serums [34]. The peptide was then commercially synthesized and used to perform phage library screening. Fortunately, one clone (no. 19) showed a significant detection of recombinant OvROPN1L but lower binding in the non-infected fecal extract. At this stage, it could be demonstrated that phages have no background interference with the binding of the OvROPN1L peptide, as the M13K07 empty phage gave no observable signal. Additionally, the polyclonal anti-rOvROPN1L cannot distinguish between recombinant OvROPN1L and non-infected fecal extract, suggesting that the polyclonal anti-rOvROPN1L cannot be used to detect *O. viverrini* infections.

scFv anti-OvROPN1L-CL19 has been successfully isolated, subcloned, and produced as a recombinant protein in an *E. coli* expression system. The scFv anti-OvROPN1L-CL19 fragment in pOPE101 was sequenced and used to predict the VH- and VL-specific domains [40]. The results indicate that scFv anti-OvROPN1L-CL19 contains specific VH and VL sequences. Moreover, the soluble scFv anti-OvROPN1L-CL19 produced from *E. coli* can specifically recognize recombinant OvROPN1L and distinguish 12 wpi coproantigens (containing OvROPN1L) from 0 wpi coproantigens with statistical significance. Compared with polyclonal antibodies against rOvROPN1L, the scFv anti-OvROPN1L-CL19 demonstrated higher titers. Low signals in the non-infected fecal extract might be caused by the subtractive biopanning that eliminated the non-specific binders from the scFv-phage library. This highlights the utility of the scFv anti-OvROPN1L-CL19. However, further research is required. Cross-reactivity to other parasites is unlikely, as the epitope OvROPN1L has already been analyzed [34].

Molecular modeling and docking demonstrated that scFv anti-OvROPN1L-CL19 is a complete antigen-binding domain, as it contains both VH and VL. The computerized prediction shows that scFv anti-OvROPN1L-CL19 likely binds OvROPN1L using both the VH and VL regions. The VH region at the CDR1, CDR2, FR1, and FR2 domains interacted with at least seven residues located on the B-cell epitope of OvROPN1L. In contrast, VL had strong binding possibilities in the CR1, CR3, and FR1 domains. This ab initio experiment corroborated the results of the wet experiments.

One limitation of this study is that the generated scFv were not analyzed with other fish-borne parasite-infected feces to verify their specificity and cross-reactivity. However, such experiments are under preparation.

## 5. Conclusions

In conclusion, in the current study, we developed a new monoclonal antibody against OvROPN1L, an *O. viverrini* sperm-specific antigen, using phage display technology. The scFv anti-OvROPN1L-CL19 is the name of our constructed single-chain monoclonal antibody that can be produced as a soluble recombinant protein in an *E. coli* expression system. ScFv anti-OvROPN1L-CL19 can bind OvROPN1L in coproantigen solution with a higher specificity than polyclonal antibodies, showing a low non-specific binding in the non-infected fecal extract. Furthermore, molecular docking strongly suggested the binding of scFv anti-OvROPN1L-CL19 with OvROPN1L. Finally, scFv anti-OvROPN1L-CL19 has significant potential to be developed as a specific early diagnostic tool for *O. viverrini* infection; however, human specimens are required for the next stage of research.

## 6. Patents

The scFv anti-OvROPN1L-CL19 generated in this study has been submitted for a patent at the Department of Intellectual Property, Ministry of Commerce, Thailand, with the submission number 2303000219.

## Figures and Tables

**Table 1:** Amino acid interactions between the OvROPN1L peptide and scFv anti-OvROPN1L-CL19.

OvROPN1L Amino Acids	scFv		Type of Interaction
	Amino Acids	Domain	
* L3	D31	VH-CDR1	H-bond
* N5	G33	VH-CDR1	H-bond
* P7	F50	VH-FR2	π-Alkyl
* D6	S52	VH-CDR2	H-bond
* D6	N53	VH-CDR2	H-bond
* P2			H-bond
* V4	L54	VH-CDR2	H-bond
* D6	S57	VH-CDR2	H-bond
* N5	G99	VH-CDR3	H-bond
* N5	G100	VH-CDR3	H-bond
* N5	N101	VH-CDR3	H-bond
* P7			H-bond
* Y8			H-bond
* H11	D135	VL-FR1	H-bond
I14	I136	VL-FR1	Hydrophobic
L24	V137	VL-FR1	Hydrophobic
L40	A143	VL-FR1	Hydrophobic
I14	S162	VL-CDR1	H-bond
P15			H-bond
* Q13	S164	VL-CDR1	H-bond
* C10	S225	VL-CDR3	H-bond

* Amino acids located in the target peptide.

## Data Availability

Not applicable.
